# Small Intestinal Bacterial Overgrowth in Children with Short Bowel Syndrome: Risk Factors, Clinical Presentation and Management—A Single-Center Experience

**DOI:** 10.3390/children12030351

**Published:** 2025-03-11

**Authors:** Maja Velimirovic, Veronika Osterman, Ana Prislan, Tadeja Pintar

**Affiliations:** 1 Department of Pediatric Surgery, Division of Surgery, University Medical Centre Ljubljana, 1000 Ljubljana, Slovenia; maja.velimirovic@kclj.si (M.V.); veronika.osterman@kclj.si (V.O.); prislan.ana@gmail.com (A.P.); 2 Services for Disease Control and Prevention, University Medical Centre Ljubljana, 1000 Ljubljana, Slovenia; 3 Department of Abdominal Surgery, Division of Surgery, University Medical Centre Ljubljana, 1000 Ljubljana, Slovenia; 4 Faculty of Medicine, University of Ljubljana, 1000 Ljubljana, Slovenia

**Keywords:** short bowel syndrome, small intestinal bacterial overgrowth, children

## Abstract

**Background:** Children with short bowel syndrome (SBS) have abnormal intestinal anatomy, secretion, or motility, which can lead to small intestinal bacterial overgrowth (SIBO). In this paper, we describe our experience with SIBO in children with SBS, focusing on potential risk factors, clinical presentation, and antibiotic treatment. **Methods:** A single-center retrospective descriptive cohort study of all episodes of clinically suspected SIBO in 16 children with SBS on home parenteral nutrition (HPN) between January 2018 and December 2022 was performed. **Results:** The mean small bowel remnant was 47 cm (SD = 31.5), with an absent ileocecal valve in 61.5% (8/13). Five children (31.2%) had at least 1 episode of clinically suspected SIBO, with a total of 25 episodes. The most common clinical presentation was diarrhea (76%), followed by meteorism (56%), loss of appetite (48%), flatulence (48%), weight loss (36%), abdominal pain (25%), and vomiting (12%). Fifty-six percent (16/25) of SIBO episodes were treated with one type of antibiotic, 36% (9/25) with two types, and 8% (2/25) with three types. Symptom resolution was achieved in 56% (14/25) of SIBO episodes after one course of antibiotic therapy. Two children (12.5%) had refractory and recurrent SIBO episodes treated with cyclic antibiotic regimens. **Conclusions:** SIBO can affect the ability of children with SBS to successfully wean off HPN. Diagnostic tests have innate challenges, and early clinical suspicion is paramount. Antibiotic therapy should be individualized considering the child’s age, gastrointestinal anatomy, and the risk of SIBO recurrence.

## 1. Introduction

Short bowel syndrome (SBS) is a complex disease that occurs after the resection of a large portion of the small or large intestine [1]. The severe reduction in the mucosal surface causes water–electrolyte disbalance, malabsorption, and malnutrition [2]. Children with SBS often develop intestinal failure (IF), defined as the reduced ability to absorb nutrients and fluids required for normal function, development, and growth [3,4]. The most common causes of extensive small bowel (SB) resection in children are perinatal and congenital diseases like necrotizing enterocolitis (NEC), malrotation with midgut volvulus, gastroschisis, and congenital intestinal atresia [5]. SBS is the cause of IF in two-thirds of cases and is the underlying disease in half of children requiring home parenteral nutrition (HPN) [3]. Factors that determine the severity of malabsorption and the prognosis of children with SBS are the length of remnant SB, the resection site of SB, the quality of remnant bowel, the length of the remnant ileum, the presence of the ileocecal valve (ICV), and the presence of intestinal continuity or a stoma [6,7]. According to some authors, the severity of IF in clinical practice can be estimated based on the amount of parenteral nutrition (PN) necessary for normal growth [8]. In addition, incretin-based therapies influence intestinal function and the ability to wean off PN. Growing evidence suggests that GLP-2 receptor agonists (GLP-2RAs) and the combination of GLP-1 and GLP-2 receptor agonists (GLP-1RA/GLP-2RA) benefit IF patients requiring PN. These therapies impact mucosal immunity, modulate endocrine cells in the terminal ileum via gut–brain mechanisms, and exert direct trophic effects on the intestinal mucosa, particularly GLP-2RA [9].

In SBS, inflammation and villous atrophy compromise the residual bowel mucosa, leading to an altered barrier function [2]. After extensive resection, the remaining small bowel dilates to compensate for ineffective propulsion, which, along with deficient nutrient absorption and motility dysfunction, causes an increase in the number of bacteria in the small intestine, leading to small intestinal bacterial overgrowth (SIBO) [10]. Other factors that also promote the development of SIBO include loss of the ICV, dependence on PN, and low gastric acidity [10]. It occurs more often in patients with impaired gut motility (intestinal atresia, NEC, gastroschisis) [11]. The excessive amount of bacteria disrupts the host physiology, causing different gastrointestinal and non-gastrointestinal symptoms and complications [12].

The most common clinical features of SIBO include abdominal distension, bloating, meteorism, weight loss, and diarrhea with malodorous stool [13]. Bacterial overgrowth can also trigger an inflammatory reaction, leading to a systemic influx of bacterial antigen–antibody complexes, consequently causing inflammatory phenomena in different organs, endotoxemia, and even sepsis [14]. The absorption of bacterial antigens, together with increased intestinal permeability, can cause bacterial translocation and activation of the nonspecific immune system leading to sepsis and hepatic damage [15]. The current literature provides limited data regarding predominant symptoms of SIBO in children with IF, especially SBS.

Treatment strategies include symptom control, hydration, nutrient supplementation, and electrolyte disbalance correction. Selective use of antibiotic therapy is recommended [16]. Studies on the effectiveness of different antibiotics for treating SIBO in children with IF of different etiology are very limited with low-quality evidence. To date, there is no study published investigating the efficacy of different types of antibiotics for treating SIBO in children with SBS. Also, no studies have defined the efficacy of rifaximin for treating pediatric SIBO, despite its favorable safety profile and proven utility in adults.

Further studies on risk factors, early symptoms recognition, and the efficacy of different antibiotics for the treatment of SIBO in children with SBS are needed to improve the management and quality of life of children with SBS. In this paper, we describe our experience with SIBO in children with SBS focusing on potential risk factors, clinical presentation, and antibiotic treatment.

## 2. Materials and Methods

### 2.1. Study Design

The study design was a single-center retrospective descriptive cohort study. All children with SBS on HPN and clinically suspected SIBO, diagnosed and managed at the University Medical Centre Ljubljana (UMCL) between January 2018 and December 2022, were included. SBS was defined as the patient’s need for HPN for more than three months.

The requirement for individual consent was waived for the retrospective study.

### 2.2. Data Collection and Variables

Patients’ demographic and clinical data were obtained from the hospital and outpatient medical records. The hospital database was retrospectively searched for codes K 91.2 and K 63.8 of the International Classification of Diseases, 10th Revision, Clinical Modification Code: postsurgical malabsorption, not elsewhere classified, and other specified diseases of intestine, respectively. Medical records were thoroughly reviewed for information regarding patients’ basic pathology diagnosed at birth, indication for surgery, resection of the ICV, the residual SB and colon length, and the presence of a stoma. Medical records were thoroughly reviewed for information on the presence of clinically suspected SIBO episodes. SIBO is traditionally defined as the overgrowth (>105 CFU/mL) of bacteria in a small intestinal aspirate [17]. In this study, the diagnosis of SIBO was based on clinical suspicion and the presence of at least two of the characteristic symptoms (diarrhea, flatulence, and meteorism). Data were collected on the most common symptoms and signs at the onset of SIBO, the choice of antibiotic treatment, and its duration. Laboratory data obtained in the acute phase of SIBO included C-reactive protein (CRP), white blood cell count (WBC), serum pH value, serum bicarbonate (HCO_3_), and base excess (BE). Bowel autonomy was defined as the ability to maintain adequate hydration, nutrition, and growth through enteral intake alone, without the need for parenteral nutrition.

### 2.3. Statistical Analysis

Descriptive statistics were used to analyze patients’ demographic and clinical data. The normality of distribution was evaluated using the Shapiro–Wilk test. Data are presented as median and quartiles, mean and standard deviation (SD), or number and percentage, as appropriate. Analyses were conducted using SPSS for Windows (v.22.0, SPSS Inc., Chicago, IL, USA).

## 3. Results

### 3.1. Patient’s Basic Pathology, Surgery and Gastrointestinal Anatomy

From January 2018 to December 2022, 16 children (56.2% female) were managed at the UMCL because of SBS. The median age at onset of SBS was 27 days (IQR 4.7–187). The median age at the end of follow up was 6.4 years (IOR 0.4–15.6). A total of 37.5% percent of the children (6/16) underwent surgery for NEC, 18.5% for intestinal atresia (3/16), 12.5% for malrotation with volvulus (2/16), 12.5% for obstructive ileus (2/16), 6.2% for gastroschisis with associated extensive bowel strangulation (1/16), 6.2% for Hirschsprung’s disease (1/16), and 6.2% for visceral myopathy with multiple bowel resections (1/16). The median age at primary operation was 27 days (IOR 4.7–187). The mean length of the remnant SB was 47 cm (SD = 31.5), with absent ICV in 61.5% (8/13). Twelve of the children (75%) had the whole colon. In three children (18.8%) the colon was absent and one (6.2%) had a 10 cm colon remnant. Ten children (62.5%) had an ileo-colonic, and three (18.7%) a jejuno-colonic anastomosis. Three children (18.7%) had an end-jejuno stoma created. All children with SBS received HPN, with 19% (3/16) receiving total HPN and 81% (13/16) partial HPN.

### 3.2. SIBO Episodes and Clinical Presentation

Of all children with SBS, 31.2% (5/16) had at least 1 episode of clinically suspected SIBO, with a total of 25 episodes (5 episodes/child, range 2–10). The most common clinical presentation was diarrhea (76%, 19/25), followed by meteorism (56%, 14/25), loss of appetite (48%, 12/25), flatulence (48%, 12/25), weight loss (36%, 9/25), abdominal pain (25%, 4/16), and vomiting (12%, 3/25). All children reported at least 2 signs or symptoms, mean 2.7 ± 1.2. A total of 60% (3/5) of children with SIBO required total HPN, and 40% (2/5) partial HPN. Eighty percent (4/5) of children with SIBO received regular therapy with a proton-pump inhibitor (PPI). None of the children with SBS and without SIBO received PPIs.

### 3.3. Laboratory Values in SIBO Episodes

The mean laboratory values were 7.2 ± 5.7 mg/L for C-reactive protein and 9.5 ± 3.9 × 10^9^/L for white blood count. The cut-off value for normal C-reactive protein was below 5 mg/L. Acidosis was present in 13% (3/23).

Table 1 presents laboratory characteristics of SIBO. Clinical suspicion of D-lactic acidosis was present in two cases (8%, 2/25).

### 3.4. Antibiotic Therapy

**Figure 1:**
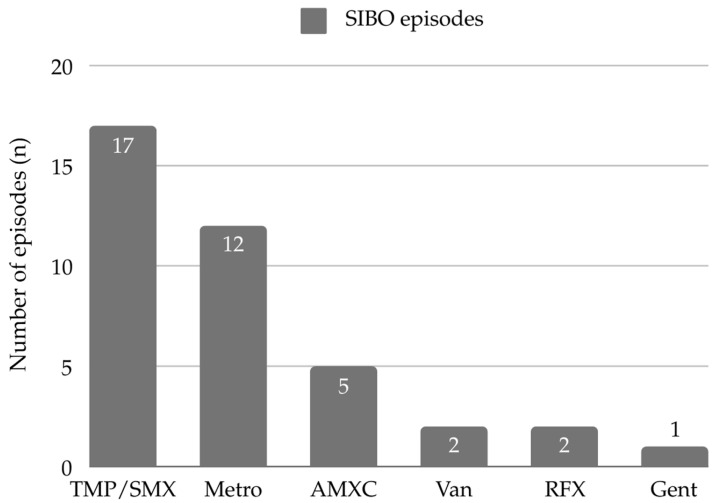
Antibiotics used in SIBO episodes. Abbreviations: AMXC—amoxicillin/clavulanic acid, Gent—gentamicin, Metro—metronidazole, RFX—rifaximin, TMP/SMX—Trimethoprim-sulfamethoxazole, Van—vancomycin.

All episodes of SIBO were treated with antibiotics. Treatment response was defined as the resolution of clinical signs and symptoms. In 56% (14/25) of SIBO episodes, a single antibiotic was used to achieve treatment response, while 36% (9/25) required two, and the remaining 8% (2/25) required three different types of antibiotics. Antibiotics were always used as monotherapy. If there was lack of treatment response, or in the case of cyclic regimen, the type of antibiotic therapy was changed. Trimethoprim-sulfamethoxazole (TMP/SMX) was prescribed most commonly (68%, 17/25), followed by metronidazole (48%, 12/25), amoxicillin/clavulanic acid (20%, 5/25), rifaximin (8%, 2/25), vancomycin (8%, 2/25), and gentamicin (4%, 1/25). The mean duration of antibiotic therapy was 11 ± 4.5 days. Figure 1 presents the different types of antibiotic therapy and the number of SIBO episodes in which they were used.

### 3.5. Recurrent/Refractory SIBO

Two (two out of five, 40%) children had refractory SIBO and required multiple courses of cyclic antibiotic therapy—one with the shortest SB remnant after bowel resection for intestinal atresia, and the other with visceral myopathy. The child with refractory SIBO and the shortest SB remnant underwent a serial transverse enteroplasty (STEP) procedure and experienced no SIBO episodes afterwards, during fifteen months of follow-up.

### 3.6. Reaching Bowel Autonomy

A total of 50% percent of all patients with SBS managed to wean off PN and achieve bowel autonomy. Of the remaining patients, 62.5% (five out of eight) had SIBO. All five children with SBS who had SIBO (five out of five, 100%) did not achieve bowel autonomy. Table 2 presents the characteristics of the five children with SIBO episodes. The most efficient type of antibiotic therapy with which resolution of symptoms was achieved is outlined in the last column.

## 4. Discussion

Different factors predispose pediatric SBS patients to a higher risk of developing SIBO. Gastroschisis with impaired gut motility and shorter residual SB remnants are risk factors for developing SIBO discussed in the literature [18]. Additionally, one study found that a longer residual SB may help prevent the development of SIBO [19]. The ICV is also an important anatomical structure maintaining intestinal integrity. In its absence, colonic bacteria can influx into the small intestine, increasing the risk of SIBO in children with SBS [20]. Studies investigating risk factors associated with SIBO were performed mostly in patients with absent ICV [21,22], and only one author found that the absence of the ICV did not predispose patients to a higher risk for SIBO. Based on this finding, the authors assumed that the physical barrier provided by the ICV is less protective, and that the concentration of the Peyer’s patch (PP) network in the terminal ileum, which plays a crucial role in both innate and adaptive immunity in the gut [17], is important in the development of SIBO [19]. M cells regulate antigen uptake and modulate systemic immune response. Dysbiosis of the intestinal microbiota not only leads to SIBO but is also associated with systemic autoimmune diseases, suggesting that PP and M cells play an important role in the induction and control of autoimmune diseases beyond mucosal immunity and response. In patients with SBS, the total mucosal surface area is reduced at the expense of the length and functionality of the small intestine, thus reducing the total mass of PP and M cells or GALTs (gut associated lymphoid tissue). These play an important role in the occurrence and incidence of SIBO, and its complications might be explained via dysregulated adaptive immune responses against potentially hostile foreign agents as well as non-harmful commensal microorganisms. Reduced mucosal surface area is directly related to the reduced presence of M cells, which have a function in transcytosis that facilitates the delivery of antigens to mononuclear phagocytes and B cells in PPs to trigger antigen-specific immune responses, and also antigen-specific S-IgA production. Reduced and inefficient S-IgA is directly related to the incidence of dysbiosis and SIBO [23]. Also, longer intestinal resections trigger dysregulation and reduced production of GLP-1, additionally related to reduced intestinal cell proliferation and intestinal adaptation after surgery. Because of diminished GLP-1 after surgery, reduced epithelial turnover and prolonged regeneration of mucosal surface area result in lengthened and inefficient intestinal adaptation, a process directly related to intestinal dysbiosis and SIBO. GLP-1 production is also related to immune response modulation, regulating mucosal T-cells and macrophages activity. Furthermore, GLP-1 is related to functional adaptation after surgery. Lengthening and improved metabolic response after GLP-1 treatment are associated with lower rates of dysbiosis and SIBO due to the influence on GALTs and might represent an important contribution to the implementation of therapeutic strategies for SBS and SIBO. Moreover, after extensive ileum resection, GLP-1 receptor agonists allow restoration of the negative feedback mechanisms and a reduction in diarrhea due to prolonged SB transit time, inhibiting hepatic bile acids production and upper gastrointestinal secretions, all related to intestinal dysbiosis and metabolic complications in SBS. Additionally, GLP-1 is co-secreted with GLP-2 in a ratio of 1:1, and the main functions of GLP-2 are stimulation of crypt cell proliferation, intestine stem cell expansion, and intestinal growth. Studies show that the extent of intestine resection in SBS patients directly correlates with loss of insulin sensitivity, impaired intestinal barrier function, low grade inflammation, and lipid accumulation. Metabolic endotoxemia, resulting from SIBO-related metabolites and dysbiosis, has been shown in observational and mechanistic studies to contribute to metabolic dysregulation and immune dysfunction [24,25].

In our cohort, all the children that developed SIBO had an absent ICV, and the highest number of SIBO episodes (10 in 5 years) was observed in a child with an absent ICV and the shortest SB remnant (30 cm), which is consistent with the results of previous studies.

The use of PPIs and a higher gastric pH have already been confirmed as a risk factor for SIBO in various studies in adults and children [19,26,27,28]. After intestinal resection, gastric secretion increases due to several factors, including the following: reduced secretion of hormones inhibiting gastric acid production, accelerated gastric emptying, and altered gut motility. The resulting dysbiosis and mucosal inflammation can further stimulate acid secretion [29]. Proximal duodenal resection more frequently corresponds to increased gastric secretions and a shorter transit time, with a consequent reduction in nutrient resorption. In distal ileum resection, shorter transit time and hyperacidity result in dysregulated B12 resorption with a consequent clinical presentation. Intestinal resections therefore frequently require treatment with PPIs and H2 blockers to suppress excessive acid production with hindered nutrient resorption [18]. In our study, eighty percent of our children with SIBO received regular therapy with a proton-pump inhibitor (PPI) and none of the children with SBS and without SIBO received PPIs, corroborating the link between a higher gastric pH and SIBO.

There are only a few studies investigating clinical manifestations of SIBO in pediatric patients, especially in patients with SBS. Clinical features during SIBO episodes can be difficult to discern from those associated with IF due to SBS. The most reported clinical feature in the literature is diarrhea [17,29]. That was also true in our cohort (76%, 19/25), with 96% (24/25) of SIBO episodes presenting with more than one clinical sign or symptom. A recent study comparing clinical features of SIBO in children with IF due to SBS (SBS-IF) and IF due to other etiologies (non SBS-IF), reported 16 episodes of clinically suspected SIBO in six children with SBS-IF (2.6 episodes/child), which is lower than in our study (5 episodes/child). In this study, children with SBS-IF also reported diarrhea as the most predominant clinical feature, especially during episodes with only one clinical sign or symptom. Therefore, in children with IF on HPN, diarrhea may be an early clinical sign of SIBO [30].

Malabsorption, bacterial translocation, and D-lactic acidosis are commonly present in children with SBS. Abundant dysbiosis is related to increased bacterial carbohydrate breakdown with elevated production of carbon dioxide, hydrogen, and methane, leading to the clinical features of bloating, pain, and abdominal distension. Reduced transit time is in direct correlation with D-lactic acidosis due to non-absorbed carbohydrates being delivered to intestinal bacteria, producing lactic acid as a by-product. Alterations of the colonic microbiota combined with an increased load of undigested carbohydrates lead to an increase in the production of D-lactate, which is absorbed and metabolized in the liver under normal anatomic circumstances [13]. Due to the inability to metabolize the large quantities of D-isomer, its toxic levels trigger D-lactic acidosis with complications [31].

In patients with SBS, disrupted enterohepatic circulation and bile acid dysregulation (downregulation of bile acid secretion and simultaneous stimulation of synthesis and uptake) lead to bile acid accumulation, bile acid toxicity, and chronic inflammation, altogether resulting in chronic liver disease and dysfunction. Extensive distal ileum resections worsen clinical presentation due to the site of bile acids resorption, presenting with steatorrhea and a wide range of nutritional deficiencies (vitamins A, D, E, K). Mandatory treatment with bile acid sequestrants could improve clinical symptoms and reduce long-term consequences alongside bile acid supplementation. On the other hand, impaired bile acids metabolism leads to intestinal inflammation caused by microbial pathogens and parenteral plant sterols, favoring dysregulation of bile acid liver metabolism via down-regulation of the nuclear fresenoid X receptor [32].

Endoscopic duodenal aspirate remains the standard method to diagnose SIBO, which is defined as a growth of >10^5^ CFU/mm^3^ of a bacterial species in the culture of luminal aspirate [18,20]. However, this technique is not well established in clinical practice, especially in children, because it is invasive and time consuming. The 2020 American College of Gastroenterology (ACG) or European guidelines recommend the use of hydrogen (H2) breath testing to diagnose SIBO in SBS patients [33,34,35]. An important limitation of this diagnostic method is that it requires active cooperation and proper adherence to instructions, making it unsuitable for patients younger than 5 years.

During SIBO episodes, the release of bacterial products and, consequently, proinflammatory cytokines into the intestinal lumen increases [36,37]. Inflammatory serum markers in our cohort of patients remained in the normal range during all SIBO episodes. CRP values were only slightly elevated with normal levels of WBC. Similarly, in studies including patients with Chron’s disease, inflammatory parameters were not significantly different in patients with and without SIBO [38,39]. These findings suggest that bacterial overgrowth is usually restricted to the small intestine and does not cause systemic inflammation [38]; therefore, serum inflammatory markers have limited value in diagnosing SIBO. This was also confirmed by our results.

The diagnosis of SIBO and treatment strategy in our patients was based on clinical presentation without the use of objective diagnostic tools. Some authors argue it is necessary to objectively diagnose SIBO before the introduction of antibiotic therapy to avoid the evolution of multidrug-resistant bacteria and opportunistic infections like *Clostridium difficile*, which occur more frequently in children with SBS [40]. The increased frequency of SIBO in SBS is related to dysbiosis, mucosal disruption, and dysregulated mucosal immunity. In children after intestinal resection, the gut microbiota is dominated by the phyla *Firmicutes* and *Proteobacteria*, both of which are proinflammatory and dysregulate gut health [31].

Antibiotics are the mainstay of treatment for SIBO, aimed at reducing the bacterial burden in the small intestine rather than eradicating it, and consequently resolving the mucosal inflammation associated with bacterial overgrowth and malabsorption. Erythromycin and amoxicillin are known to have propulsive efficacy and have been used to alleviate gut dysmotility. They may be useful for treating SIBO because of their propulsive effect and ability to remodel the microbiome [2]. Their efficacy for treating SIBO has not yet been studied in pediatric patients with SBS. In one child in our cohort with SBS and visceral myopathy, amoxicillin/clavulanic acid was used in a cyclic regimen because of refractory SIBO and recurrent episodes. We assume its effectiveness in treating SIBO symptoms in this child with severe gut dysmotility was predominantly due to its prokinetic properties.

Rifaximin is a non-absorbable antibiotic, and it is efficient against both Gram-positive and Gram-negative strains of aerobic and anaerobic bacteria [2,41]. Its efficacy has also been proven in treating SIBO in children with irritable bowel syndrome [42]. Rifaximin is an effective and safe antibiotic often used for the treatment of SIBO in adults [43,44,45]. It has an excellent safety profile due to its lack of systemic absorption [43,44]. According to published reports, rifaximin is well tolerated in adults with only a few adverse effects such as nausea, gastrointestinal irritation, fatigue, peripheral edema, dizziness, and muscle spasm [44]. However, there is considerably less consensus on the use of rifaximin in children, especially in children with SBS [45]. The few available studies in children have various limitations, including a small number of patients and a lack of control or subgroups with specific underlying diseases [16]. Due to a general lack of data, its use is recommended in children older than eight years [46]. Furthermore, studies have shown diverse results concerning the efficacy of rifaximin in children with SIBO [16]. In our clinical practice we have observed a mixed clinical response to rifaximin with no significant clinical improvement of SIBO in some patients. Some children tolerated the drug poorly and it was discontinued. Two (two out of five, 40%) of our children reported nausea and vomiting, and one (one out of five, 20%) had a significant increase in stool losses. In children with a poor tolerance of rifaximin, we have used other antibiotics to treat SIBO, such as trimethoprim-sulfamethoxazole, metronidazole, or amoxicillin-clavulanic acid.

A recent study in children with SIBO and different underlying pathologies did not find any difference in the efficacy between metronidazole, rifaximin, and other antibiotics in treating SIBO symptoms [47]. Furthermore, although initial antibiotic treatment was effective in most children, 22.2% of them still needed one or more repeat courses of antibiotics, suggesting a high SIBO treatment failure or recurrence rate [47].

In our cohort, two (two out of five, 40%) children developed recurrent and refractory SIBO. One child with the shortest SB remnant after resection for intestinal atresia, received cyclic antibiotics for refractory SIBO: one week of metronidazole followed by one week of pause, followed by one week of TMP/SMX. The antibiotic cycle was repeated for three months until resolution of symptoms for every SIBO episode. Ultimately, the child underwent a STEP procedure and did not experience any SIBO episodes afterwards in the follow-up period. Another child with visceral myopathy and severe gut dysmotility received amoxicillin/clavulanic acid for ten days every month when there was SIBO recurrence with clinical manifestation. Patients with recurrent SIBO can become dependent on antibiotic therapy cycles and can develop infections with multidrug-resistant bacterial strains. Recent studies investigating the structure of the gut microbiota in pediatric patients with SBS confirmed important gut dysbiosis with an abundance of pathogenic intestinal strains due to antibiotic therapy [48,49].

PN has greatly enhanced the life expectancy of children with SBS, reducing mortality and improving overall outcomes. However, prolonged use of PN is linked to several complications, such as liver disease, catheter-related issues, and bloodstream infections [10]. In our study, we evaluated the outcome of weaning from PN, which is a critical milestone in the management of children with SBS. Fifty percent of children with SBS in our cohort successfully weaned off PN and achieved bowel autonomy, highlighting the potential for recovery in a subset of SBS patients. However, our findings also underscore the challenges faced by children with SBS and SIBO, as a significant portion (62.5%) of those who did not achieve bowel autonomy had concomitant SIBO. Among the children with SBS and SIBO, none achieved bowel autonomy or successfully weaned from HPN. These results suggest that the presence of SIBO likely exacerbates the malabsorption and gastrointestinal dysfunction commonly observed in SBS, which may hinder the ability to transition from PN to enteral feeding and achieve bowel autonomy. Our study’s findings are consistent with prior research, which found a statistically significant association between successful weaning from PN and factors such as the length of the SB remnant (*p* = 0.002) and the presence of ICV (*p* < 0.001) [50]. Both factors have also been identified as risk factors for SIBO, further linking them to the challenges of PN weaning in children with SBS [10,19,20]. The presence of SIBO in our cohort may therefore be a critical factor hindering the transition from PN to enteral feeding, emphasizing the importance of managing such complications in facilitating enteral autonomy.

The important limitations of our study are its retrospective design, small number of children included, and great variability in their characteristics. The diagnosis of SIBO and evaluation of treatment response were based on clinical symptoms, therefore our results lack objectivity, and the reported data in the existing literature in the pediatric population is also largely based on the clinical features of the disease. Further prospective studies on the effectiveness of different antibiotics for treating SIBO in children with SBS using objective diagnostic tools and implementation of all the therapeutic modalities are needed to improve the management of SIBO in children with SBS and reduce long-term complications.

While most of the available literature addresses SIBO in general populations [13,14,16,20,27,28,31,35,39,40], there is a notable lack of high-quality evidence and comprehensive studies specifically focusing on SIBO in children with SBS [18,19,30]. As a result, the correlation between SIBO and SBS remains underexplored, with gaps in understanding the impact of SBS-specific factors such as malabsorption, dysbiosis, and altered transit time on SIBO development. Despite the small, single-center nature of our study, it provides valuable insights into an area with limited existing data. By identifying risk factors, clinical presentations, and treatment approaches, our findings contribute to a better understanding of this complex condition and may serve as a foundation for future multi-center studies and clinical guidelines. The rarity of SBS and the restrictions of SIBO diagnostics in children make large studies challenging, further underscoring the importance of our dataset in improving patient care and guiding future research.

## 5. Conclusions

Children with SBS are at high risk for SIBO, which can affect their ability to successfully wean off HPN. It is recommended to diagnose SIBO with objective tools before antibiotic therapy. However, diagnostic tests have innate challenges in children, and a strong index of clinical suspicion is paramount. To date, there are no evidence-based recommendations for specific antibiotic regimens that should be used in the treatment of SIBO in children with SBS. Decision-making regarding antibiotic therapy should be individualized based on a patient’s age and underlying pathology, considering possible motility disorders and gastrointestinal anatomy, which can vary greatly in different patients with SBS due to different surgical interventions and altered intestinal and liver physiology. The risk for prolonged antibiotic therapy and SIBO recurrence should also be considered.

## Figures and Tables

**Table 1:** Laboratory values.

Variable	Measured Number of Values	Total N = 25
CRP, mean ± SD, mg/L	25	7.2 ± 5.6
WBC, mean ± SD, ×10^9^/L	25	9.5 ± 3.9
Hb, mean ± SD, g/L	24	108.1 ± 15.2
Fe, mean ± SD, µmol/L	18	11.3 ± 4.5
Acidosis, *n* (%)	23	3 (13%)
pH, mean ± SD	23	7.37 ± 0.05
HCO_3_, mean ± SD, mEq/L	23	22.9 ± 3.4
BE, mean ± SD, mmol/L	23	−2.3 ± 3.4

Abbreviations: BE—base excess, CRP—C-reactive protein, Fe—serum iron, Hb—hemoglobin, HCO_3_—serum bicarbonate, *n*—number, SD—standard deviation, WBC—white blood count.

**Table 2:** Children with SBS and SIBO.

Basic Pathology	SB Length (cm)	ICV (Y/N)	Stoma (Y/N)	PN (PPN/TPN)	Colon Absent (Y/N)	No. of SIBO Episodes	Efficient ATB Therapy
Intestinal Atresia	30	N	N	TPN	N	10 (recurrent)	Metro, TMP/SMX
Visceral Myopathy	40	N	Y	TPN	Y	5 (recurrent)	AMXC
Gastroschisis	NA	N	Y	TPN	Y	2	AMXC
Hirschsprung’s disease	120	N	Y	PPN	Y	2	Metro, TMP/SMX
Midgut Volvulus	80	N	N	PPN	N	6	Metro, TMP/SMX

Abbreviations: AMXC—amoxicillin/clavulanic acid, ATB—antibiotic, ICV—ileocecal valve, Metro—metronidazole, NA—not applicable, N—no, PN—parenteral nutrition, PPN—partial parenteral nutrition, SB—small bowel, SIBO—small intestinal bacterial overgrowth, TMP/SMX—trimethoprim-sulfamethoxazole, TPN—total parenteral nutrition, Y—yes.

## Data Availability

The raw data supporting the conclusions of this article will be made available by the authors on request.
